# Differential susceptibility to plasticity: a 'missing link' between gene-culture co-evolution and neuropsychiatric spectrum disorders?

**DOI:** 10.1186/1741-7015-10-37

**Published:** 2012-04-17

**Authors:** Rachel Wurzman, James Giordano

**Affiliations:** 1 Interdisciplinary Neuroscience Program, Georgetown University Medical Center, 4000 Reservoir Rd, Washington, DC, 20007, USA; 2 Center for Neurotechnology Studies, Potomac Institute for Policy Studies, 901 N. Stuart St, Arlington, VA, 22203, USA; 3 Department of Electrical and Computational Engineering, University of New Mexico, ECE Bldg 46, 1 University of New Mexico Dr., Albuquerque, NM, 87141-0001, USA; 4 Human Science Center, Ludwig-Maximilians Universität, Goethestraβe 31, D-80336, Munich/Professor Max-Lange Platz 11 83646, Bad Tölz, Germany

## Abstract

Brüne's proposal that erstwhile 'vulnerability' genes need to be reconsidered as 'plasticity' genes, given the potential for certain environments to yield increased positive function in the same domain as potential dysfunction, has implications for psychiatric nosology as well as a more dynamic understanding of the relationship between genes and culture. In addition to validating neuropsychiatric spectrum disorder nosologies by calling for similar methodological shifts in gene-environment-interaction studies, Brüne's position elevates the importance of environmental contexts - inclusive of socio-cultural variables - as mechanisms that contribute to clinical presentation. We assert that when models of susceptibility to plasticity and neuropsychiatric spectrum disorders are concomitantly considered, a new line of inquiry emerges into the co-evolution and co-determination of socio-cultural contexts and endophenotypes. This presents potentially unique opportunities, benefits, challenges, and responsibilities for research and practice in psychiatry.

Please see related manuscript: http://www.biomedcentral.com/1741-7015/10/38

## Introduction

The diathesis stress model for genetics of psychopathology contends that individuals with certain genes may possess greater 'vulnerability' to dysfunction when exposed to adverse environmental conditions than individuals lacking these genes. While this theory has predominated psychiatric research and practice, it remains puzzling, given evolutionary theory, that natural selection would favor allelic variants that increase vulnerability to adversity. In this issue, Brüne offers a possible solution to this conundrum by reconsidering so-called 'vulnerability' genes, and suggesting that they be regarded as genes that confer greater susceptibility to plasticity, rather than pathology [1]. We applaud this approach and agree with his assertion that this elevates the importance of environmental variables in determining developmental outcomes. Here, we advance this discussion to encompass implications for psychiatric nosology and the potential for more dynamic interactions between genes and culture with respect to psychological function and dysfunction.

Brüne provides an overview that argues for '... an association of genetic plasticity with both behavioral and neuroanatomical correlates of gene-environment interaction' [1]. Brüne supports the proposal of Belsky and co-workers that genes associated with increased vulnerability to psychopathology under adverse environmental conditions are also likely to confer advantages in the same domain of psychological functioning in supportive or enriched environments [2-4]. Brüne asserts that '... it may be more accurate to speak of differential susceptibility or plasticity conferred by genetic variation, that is responsivity to both positive and negative conditions, rather than focusing one-sidedly on vulnerability' [1]. While noting that additional studies will be required to more fully address and resolve extant questions, Brüne claims that the '... diathesis-stress model of psychopathological conditions needs to be refined, last, but not least, by anchoring clinical research on gene-environment interaction in evolutionary theory' [1]. The explanatory power of this shift in emphasis is most obvious in its resolution of the apparent paradox in positive selection of allelic variants that increase vulnerability to adversity, without necessarily requiring that compensatory advantages be concomitantly conferred in an unrelated functional domain (aka 'balanced polymorphisms'). Rather, it allows for such compensatory balance to occur within a single functional realm, thereby supporting that function in a given behavioral or psychological domain exists along a continuum.

## The concept - and utility - of spectrum disorder

When attempting to define psychopathology (particularly in a broadly cultural context), there is often ambiguity about where to establish the boundary between 'normal' and 'abnormal' function. Interestingly, the shift from discrete or binary classifications of 'normal' *vs*. 'abnormal' to one of a continuum along functional domains is also a prerequisite for the concept of neuropsychiatric spectrum disorders [5,6]. In this regard, we are particularly enthusiastic about the apparent complementarity of Brüne's 'differential plasticity' interpretation of susceptibility genes, and nosological models of putative neuropsychiatric spectrum disorders. We have previously argued in favor of grounding neuropsychiatric syndromes to underlying biological factors using a spectrum nosology that considers 'particular genotypic factors' to 'predispose endo- and exophenotypes that are differentially expressed through interaction(s) with internal and external environmental influences throughout the lifespan' [6,7]. Specifically, we asserted that rather than seeking biological mechanisms for genetic and biochemical causes to 'exert their influences uni-directionally' towards a discrete organic disorder, it may be more useful to conceive of abnormality as a progressive loss of non-linear adaptive properties within and between particular brain networks, which could manifest as a spectrum of possible effects from the cellular to the cognitive-behavioral and even socio-cultural levels [6,7]. In this way, we proposed that given the lack of clear biological mechanisms for causal variables leading to a shift from normal to abnormal functioning, such a spectrum concept might bridge the 'missing link between the medical model and psychiatry', and be useful to reconcile ontological assumptions of (neurobiological) essentialism in the medical model of psychiatry and associated limitations in nosological constructs.

Accordingly, the notion of putative 'plasticity genes' as presented by Brüne is important to strengthen our position and to fortify the medical model of psychiatry (as defined by the ontological assumptions of realism, naturalism, reductionism, and essentialism) [7]. Inasmuch as evidence concerning genetic factors' contribution to the prevalence of psychiatric disorders is interpreted as validating these disorders as discrete entities with organic etiologies, the 'missing link' we refer to is the unmet need to relate essential biological mechanisms (that cause putatively-unified, organic disease states) to psychiatric nosological constructs of heterogeneous symptom clusters (that are often validated post-hoc) [7,8]. The potential to link genetic factors to differences in neuroanatomical substrates - as well as behavior - enables a strongly correlational, if not causal interpretation of these structure-function relationships, despite the fact that signs and symptoms from heterogeneous functional domains nevertheless tend to cluster in certain disorder domains [9]. Importantly, both models call for a methodological shift in gene-environment-interaction studies to redefine the 'good' end of the continuum of environmental-exposure and psychological function as positive, rather than being merely absent adversity and/or disorder. This emphasizes environmental contexts - inclusive of socio-cultural variables - as mechanisms that contribute to clinical presentation.

## A role for 'culture'

It is here that we believe the most profound impact of this theory becomes apparent. A significant implication is that if such genes and socio-cultural environmental variables co-determine the degree of susceptibility to plasticity, then these cannot - and should not - be considered independently as regards their role in establishing predispositions to maladaptive neurological function and associated psychological sequelae. Given the role of oxytocin in cognition and the importance of neuro-cognitive/environmental interactions to the evolution of human culture(s) and society [10-12], observations that allelic variation of an oxytocin receptor (OXTR) confers variable susceptibility to psychopathology has consequences for an understanding of potential relationships between the evolution of culture and a regional, population-based distribution of genotypes. Perhaps most importantly, that a given OXTR allelic variation confers differential degrees of susceptibility to both positive and negative plasticity in distinct genotypes, depending upon the extent of adversity or favorability of early experience, allows for a reinterpretation of the (bi-directional) interactions of culture and genetics in the determination of psychological function *vs*. dysfunction. While Brüne notes that from an evolutionary perspective, traits associated with the allelic variation of OXTR may confer a selective advantage if environmental contingencies are beneficial, he stops short of explicitly considering the role of culture in contributing to the valence of environmental variables and the effects of early experience.

Yet, suggesting that the importance of socio-cultural environmental variables is relative with respect to the effects of OXTR allelic variation on psychological function (depending on ethnicity) leads to questioning whether a given socio-cultural environment may confer particular advantages or disadvantages depending on genotype. However, since culture is more readily adapted than genetics, implicit in the shift from 'vulnerability/stress-diathesis' to 'differential susceptibility' and its accompanying allowance for the possibility of benefit in the same axis, is some form of 'gene-culture co-evolutionary theory' [13-15]. The latter originally posited that culture may have evolved with selective pressures upon genotypes that favor behaviors aligned with advantageous cultural values, for example collectivism in regions of high pathogen prevalence [16]. The reciprocal relationship between genes and (individual *vs*. collective) cultural values was explored by Chiao and Blazinsky (2009) in the context of one of Brüne's candidate 'plasticity genes', the serotonin transporter functional polymorphism (5-HTTLPR) [17]. In accordance with Brüne's 'differential susceptibility' model, their interpretation emphasized that cultural values may have been adapted to reduce the exposure of a population's members to 'environmental pathogens' such as chronic life stress, given the prevalence of a genotype associated with increased vulnerability to psychopathology [17]. However, Brüne's consideration of the potential for positive plasticity provides a means for a culture to evolve to optimally suit a population's prevalent genotype(s). For example, it is possible that over evolutionarily-relevant time spans, aspects of cultures may have been positively selected that encouraged development of a beneficial endophenotype (for example, amygdala size with the OXT allele) to enable a significant proportion of the population to flourish [9,18].

## Relevance and importance to psychiatry

The implications of genetic variation conferring plasticity - as opposed to vulnerability - not only reinforce the importance of recognizing dynamic bio-psychosocial models, but suggest the need to reconsider the relative role(s) of culture when seeking to understand how disorders are manifest. Perhaps most important is the need to do so in a way that does not assume that one set of cultural/environmental variables are suitable for the entire range of diverse genotypes and endophentotypes encountered. Beyond cultural relativism, the enormity of potential combinations of gene combinations and environments calls for psychiatry to account for individual differences according to an individual's own array of factors (for example some form of 'personalized medicine'), and still retain something akin to group validity and/or generalizability. We posit that concepts of bio-psychosocial spectrum disorders are well-suited to this. However, we also advocate caution against assuming that one treatment model is optimal for individuals grouped either by genotype or culture, because individual endophenotypes likely depend upon complex interactions of these (genetic and environmental) factors.

Therefore, when regarding both models of susceptibility to plasticity, and neuropsychiatric spectrum disorders, a new line of inquiry emerges into the co-evolution and co-determination of socio-cultural contexts and endophenotypes, and further, into the consequences of displacing bio-psychological characteristics to various socio-cultural milieu [19]. This is particularly relevant since, as Brüne notes, the medical model of psychiatry is becoming increasingly popular and utilized in non-Western countries where differing socio-cultural 'set points' may accommodate the prevalent regional endophenotypes. Moreover, while human genetic traits and culture have evolved for tens of thousands of years, the large-scale socio-cultural shifts of the past century have made it difficult to account for, much less predict, how changes in environments and social structures will affect neuropsychiatric function. These shifts in environmental and socio-cultural variables are occurring on a global scale, and not all genotypes and endophenotypes will be equally susceptible to potential advantages and/or deprivations disposed or evoked by such rapid and radical change.

We argue that this compels the need to understand concepts, constructs, and effects of norms in a more complex and relative sense; else there is risk of psychiatric ethnocentrism incurred by conflating values that contribute to optimum functioning of one particular population with 'universally favorable' values. This would have detrimental consequences for individuals who may be better suited to a different approach to their bio-psychosociocultural contexts, as it is becoming clear that neurobiological diversity is desirable in the face of challenges presented by cultural shifts (in family structure, communication, social support, the volume and manifestations of ambient sensory stimuli, economics, and so on) [20-22]. So, while stress-diathesis, vulnerability-centered models of psychiatric disorders may have utility in understanding how these socio-cultural and neurobiological variables interact to generate disease, disorder, and dysfunction, as Brüne explains, it is also necessary to consider the good as more than merely an absence of adversity and disorder if the goal is to facilitate true flourishing, and not just the mitigation of suffering.

## Conclusions: addressing benefits and responsibilities

A unique and valuable opportunity is thus afforded by adopting the differential susceptibility-to-plasticity model of psychiatric disorders. We opine that an enhanced understanding of neurobiopsychosocial mechanisms contributory to neuropsychiatric diversity would shift the focus in psychiatry toward: (1) better identifying what, how, and why complex variables may be important to epidemiological patterns and trends of certain conditions (for example, autism; attention deficit); (2) perceiving the potential benefits afforded by those factors that increase susceptibility to neural plasticity upon exposure to environmental contingencies, and where they might, under given circumstances, present an evolutionary advantage or disadvantage; and (3) distinguishing relevant bio-psychological and social variables upon which to intervene in order to promote a fuller expression of potential benefits in individuals with such predispositions.

Therefore, it follows that opportunities arise not only for diagnostic taxonomy and nosology (and accordingly, for research and scientific knowledge), but for a more finely-grained understanding of the dynamic interaction between culture (herein defined as both a medium and forum for respectively generating and fostering biological and socio-environmental characteristics) and human mental - if not overall - health. This incurs a number of ethical, legal, and sociological implications - and responsibilities - for research, treatment approaches, and the positive or negative influence of psychiatry in promoting human wellbeing. Further, and albeit optimistically, such knowledge might prompt an attitudinal shift that could be instrumental in calming the fears of those who feel threatened and alienated by the medical model of psychiatry, without having to sacrifice an understanding of psychological (dys)function as being grounded to neural substrates.

## Competing interests

The authors declare that they have no competing interests.

## Authors' contributions

RW and JG collaborated to develop the literature review and core concepts of this commentary, and co-wrote the body of the text. Both authors read and approved the final manuscript.

## Authors' information

RW is a doctoral candidate in the Interdisciplinary Program in Neurosciences at the Georgetown University Medical Center, Washington, DC, USA, and is a Resident Scholar of the Center for Neurotechnology Studies of the Potomac Institute for Policy Studies, Arlington, VA, USA. JG is Director of the Center for Neurotechnology Studies of the Potomac Institute for Policy Studies, Arlington, VA, USA; Fulbright Gast Professor of Neuroscience, Neurotechnology and Ethics at the Human Science Center of Ludwig-Maximilians Universität, Munich Germany, and Research Professor of Neurosciences and Ethics in the Department of Electrical and Computational Engineering, University of New Mexico, Albuquerque, NM, USA.

## Pre-publication history

The pre-publication history for this paper can be accessed here:

http://www.biomedcentral.com/1741-7015/10/37/prepub
